# Phase retrieval in holographic data storage by expanded spectrum combined with dynamic sampling method

**DOI:** 10.1038/s41598-023-46357-9

**Published:** 2023-11-02

**Authors:** Ruixian Chen, Jianying Hao, Jinyu Wang, Yongkun Lin, Kun Wang, Dakui Lin, Xiao Lin, Xiaodi Tan

**Affiliations:** Information Photonics Research Center, College of Photonic and Electronic Engineering, Key Laboratory of Opto-Electronic Science and for Medicine of Ministry of Education, Fujian Provincial Key Laboratory of Photonics Technology, Fujian Provincial Engineering Technology Research Center of Photoelectric Sensing Application, Fujian Normal University, Fuzhou, 350117 China

**Keywords:** Optical data storage, Imaging and sensing

## Abstract

Phase retrieval in holographic data storage by expanded spectrum combined with dynamic sampling method is proposed, which serves to both reduce media consumption and to shorten the iterative number of phase code retrieval. Generally, high-fidelity phase retrieval requires twice Nyquist frequency in phase-modulated holographic data storage. To increase storage density, we only recorded and captured the signal with Nyquist size and used the frequency expanded method to realize high-fidelity phase retrieval. In the decoding process, the iterative Fourier transform algorithm is used to retrieve the phase information of the reconstructed beam. The expanded spectrum is dynamically sampled, which can provide a faster convergence path for the phase retrieval. We aimed to demonstrate the possibility of integrating various methods on the Fourier domain and providing a potential way to improve the performance of holographic data storage systems. The simulation and experimental results proved the combination of processing methods in frequency spectrum was benefit.

## Introduction

The international authority Statista predicts that the global data generation will reach 175 Zettabytes in 2025^1^. The data amount is increasing in the explosive way. Although the development of traditional magnetic storage is very mature, its storage capacity is only increased by about 20% every year, which is obviously unable to meet the speed of data growth. In addition, magnetic storage is more suitable for hot data storage due to its short service life and high energy consumption in the process of data maintenance. But for large proportion cold data, optical storage is more suitable with a longer service life and lower energy consumption. However, traditional optical storage such as Blu-ray disc is almost reaching the limitation of theoretical density based on the two-dimensional surface storage mode^2^. Holography can be used in fields such as storage, imaging, and metasurfaces^3–6^. Holographic data storage brings a new development of optical storage by using a 3-dimensional volume storage mode and 2-dimensional data transmission^4, 7, 8^. It is one of most powerful candidates of the new generation of data storage technologies^9–14^.

The holographic data storage technology records the amplitude and phase of the signal beam in the media in the form of a hologram. The encoding and decoding approaches of traditional holographic data storage technologies use amplitude modulation, which ignores phase information^15, 16^. The amplitude encoding has a relatively low encoding rate, and when the recording media is placed near the back focal plane of the lens to increase the recording density, the focused amplitude-encoded beam becomes excessively concentrated. This phenomenon not only decreases the signal-to-noise ratio (SNR) but also consumes more media. However, phase encoding itself is a form of phase modulation that ensures uniform energy distribution in the media, improving both the encoding rate and SNR^17–19^. The phase distribution cannot be detected by CMOS directly, but it can be decoded by a variety of decoding methods, such as phase shifting interferometry, code pair phase shifting interferometry, transport of intensity equation (TIE), iterative Fourier transform algorithm (IFTA), etc.^20–25^. From the perspective that the holographic data storage system requires compact, stable and simple, the single-shot IFTA is more appropriate. In phase-modulated holographic data storage, IFTA combined with embedded data can be used to significantly decrease the iterative numbers of phase retrieval^26^. There is still a space for further improvement in phase retrieval efficiency by providing a better convergent path, known as dynamic sampling^27^. To ensure the accuracy of phase retrieval, more high-frequency components need to be recorded on the media when using complex multi-level phase images in phase-modulated holographic data storage. Generally, high-fidelity phase retrieval requires at least twice the Nyquist frequency information. The more information recorded, the more media consumption. Media consumption refers to the utilization or occupation of the recording media's space by the recorded information. Once information is recorded in the media, the corresponding region is considered consumed. Therefore, in order to increase storage density, we only recorded and captured the signal with Nyquist size and used frequency expanded method to achieve high-fidelity phase retrieval^28^. In this paper, we combined the frequency expanded method and dynamic sampling method to prove the possibility of integration of multiple treatment methods on the Fourier domain, and achieved the phase retrieval with less media consumption, lower bit error rate (BER) and shorter iterative numbers.

## Methods

**Figure 1:**
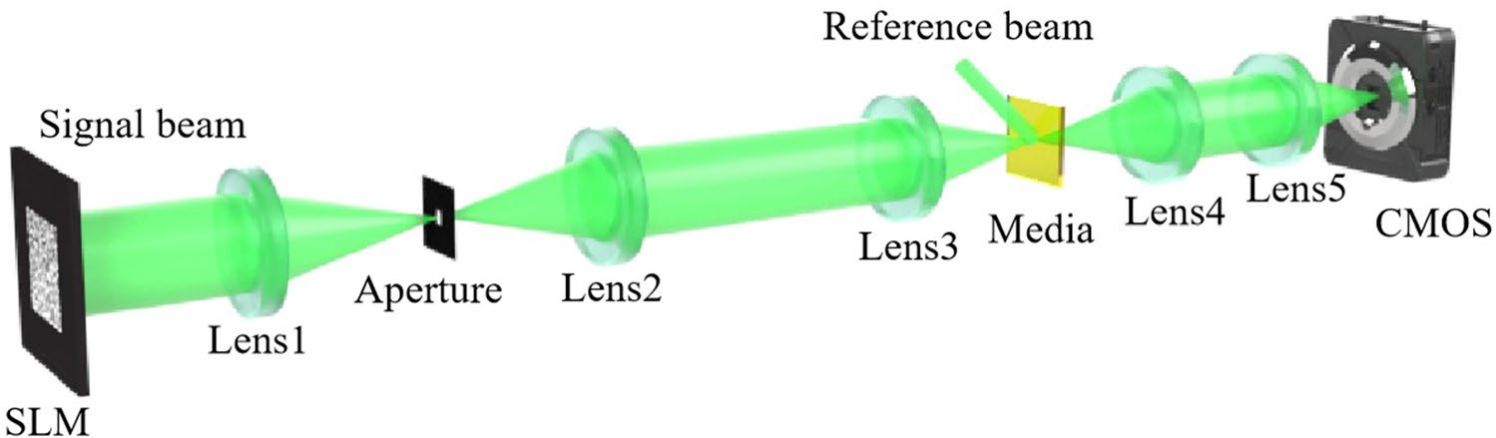
A scheme of the phase-modulated holographic data storage system.

A scheme of the phase-modulated holographic data storage system is shown in Fig. 1. A spatial light modulator (SLM) generates a signal beam according to the phase data page. In the writing process, Lens1 and Lens2 form a 4*f* system, where Lens1 focuses the light onto the aperture, which filters out certain high-frequency components. The remaining information is then imaged onto the focal plane of Lens2. The signal beam is converged by Lens3 and interferes with a reference beam to record hologram in the media. To reduce media consumption, the recording area is limited by a Nyquist size aperture, and the aperture works as a low-pass filter. The aperture refers to the physical aperture or opening through which the light passes in the holographic system. In the reading process, a same reference beam illuminates the hologram in the media to form a reconstructed beam. The reconstructed beam is formed at the back focal plane of the Lens4. Finally, the reconstructed beam passes through a Fourier transform lens (Lens5). The single-shot Fourier intensity distribution of the reconstructed beam, which is the only source for phase retrieval by iterative computing, is captured by the CMOS detector placed at the back focal plane of the Lens5. In the decoding process, we use the frequency expanded method first to build a new spectrum owning more high-frequency components and use the dynamic sampling method to provide a faster convergence path for the phase retrieval.

### Combined the frequency expanded method and the dynamic sampling method

Holographic data storage can increase storage density by different multiplexing technologies such as wavelength multiplexing, angular multiplexing, shift multiplexing, potential polarization-multiplexing and so on^29–33^. In addition, reducing the recording area of data page is also an effective approach for improving storage density^34^. The smaller the recording area is, the higher the storage density is. Generally, the aperture size is limited to approximately 2 times Nyquist size to ensure that the reconstructed beam will not be distorted in the phase-modulated holographic data storage, and the Nyquist size *w* can be defined by Eq. (1)^12^,

1 $$w = \frac{\lambda f}{d},$$

where λ denotes wavelength, *f* and *d* denote the focal length of lens and space width of one phase data on the SLM respectively.

**Figure 2:**
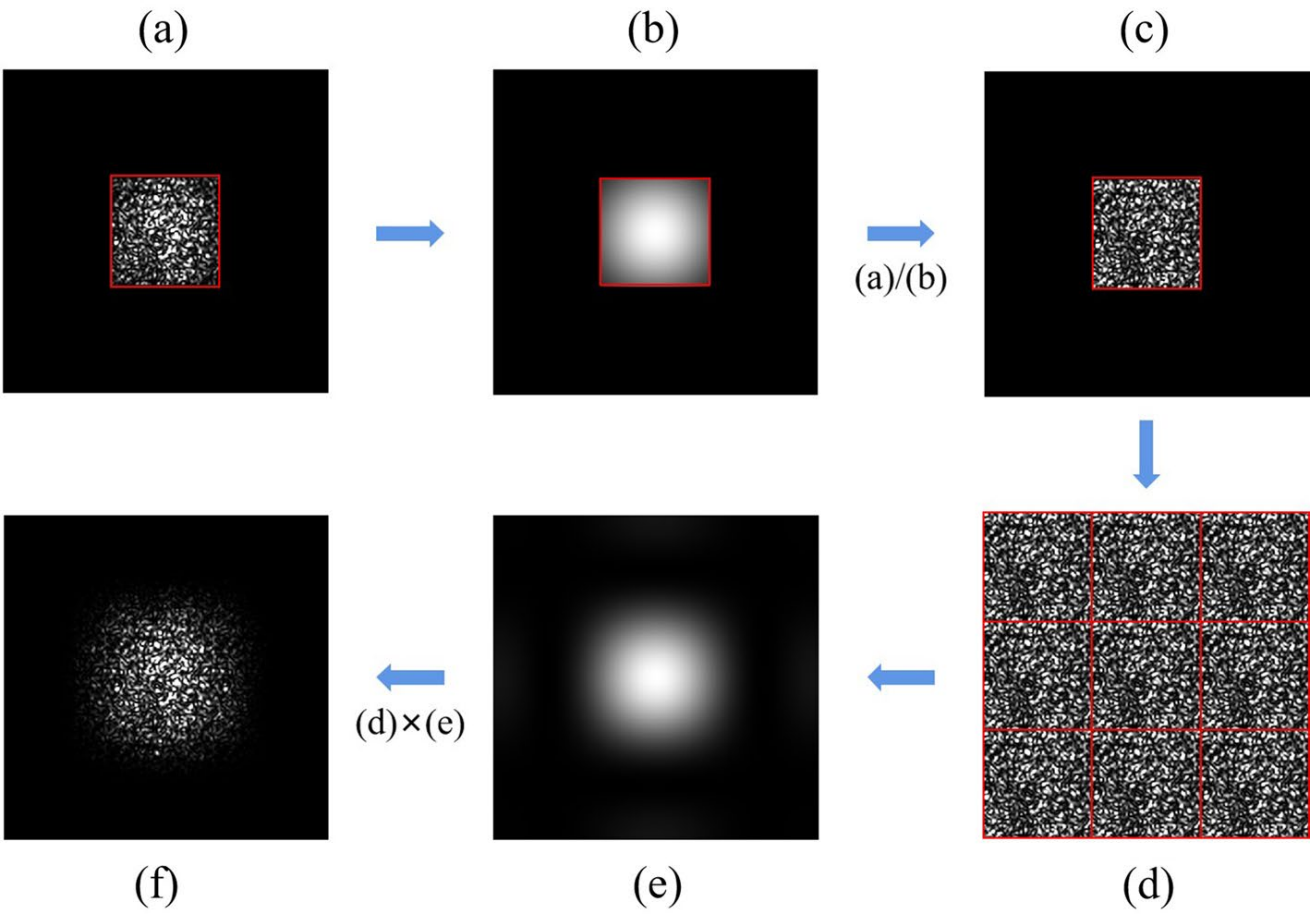
The frequency expanded process. (**a**) The Fourier intensity with Nyquist size, (**b**) the Fourier intensity of the rectangular window with Nyquist size, (**c**) the normalized Nyquist frequency, (**d**) the preliminary expanded Fourier frequency, (**e**) the Fourier intensity of the rectangular window with 3 times Nyquist size, (**f**) the final expanded Fourier intensity.

The frequency expanded method only requires recording the signal with Nyquist size, which reduces media consumption and improves recording density. The frequency expanded process is shown in Fig. 2. All the red boxes represent Nyquist size. Figure 2a shows the Fourier intensity with Nyquist size captured by CMOS. The Fourier intensity can be understood as the frequency distribution multiplied by their corresponding intensity distribution. The frequency distribution is periodic with the Nyquist interval, meaning that the spectrum with Nyquist size already contains the information required for frequency expansion. In the simulation, each phase is represented by 4 × 4 pixels on the SLM, and a single SLM pixel has dimensions 20 μm × 20 μm, then the space width of one phase data in the SLM is 80 μm. The size of each phase on the SLM, represented by a matrix, is referred to as a rectangular window. The intensity distribution corresponding to the rectangular window in the simulation calculation is similar to the envelope of the actual more complex Fourier intensity distribution^27^. Through simulation, the Fourier spectrum corresponding to the rectangular window is completely known. Therefore, an approximate intensity distribution can be obtained. However, there are discrepancies between the simulated Fourier intensity distribution corresponding to the rectangular window and the actual more complex Fourier intensity distribution. This introduces error noise during the frequency expansion process. The Fourier intensity of the rectangular window with Nyquist size and 3 times Nyquist size are shown in Fig. 2b,e, respectively. The normalized Nyquist frequency shown in Fig. 2c can be obtained by dividing the Fourier intensity of Fig. 2a by the Fourier intensity of Fig. 2b. The normalized Nyquist frequency is obtained by performing intensity normalization on the Fourier spectrum with Nyquist size. The frequency distribution has a periodicity of the Nyquist interval, which means that the normalized Nyquist frequency can be periodically copied and pasted. Figure 2d shows the preliminary expanded Fourier frequency for the normalized Nyquist frequency. The final expanded Fourier intensity shown in Fig. 2f is obtained by multiplying the expanded Fourier frequency of Fig. 2d with the Fourier intensity distribution of Fig. 2e. We can see that artificially completed high-frequency components are present in the expanded Fourier frequency. We can extend the Nyquist frequency to any size by the frequency expansion process shown in Fig. 2. Generally, a larger extended frequency spectrum means more high-frequency components. However, more high-frequency components do not necessarily result in better phase retrieval performance due to the poor noise resistance of high-frequency components. In the following simulations, we will discuss how to choose the appropriate size for frequency expansion.

**Figure 3:**
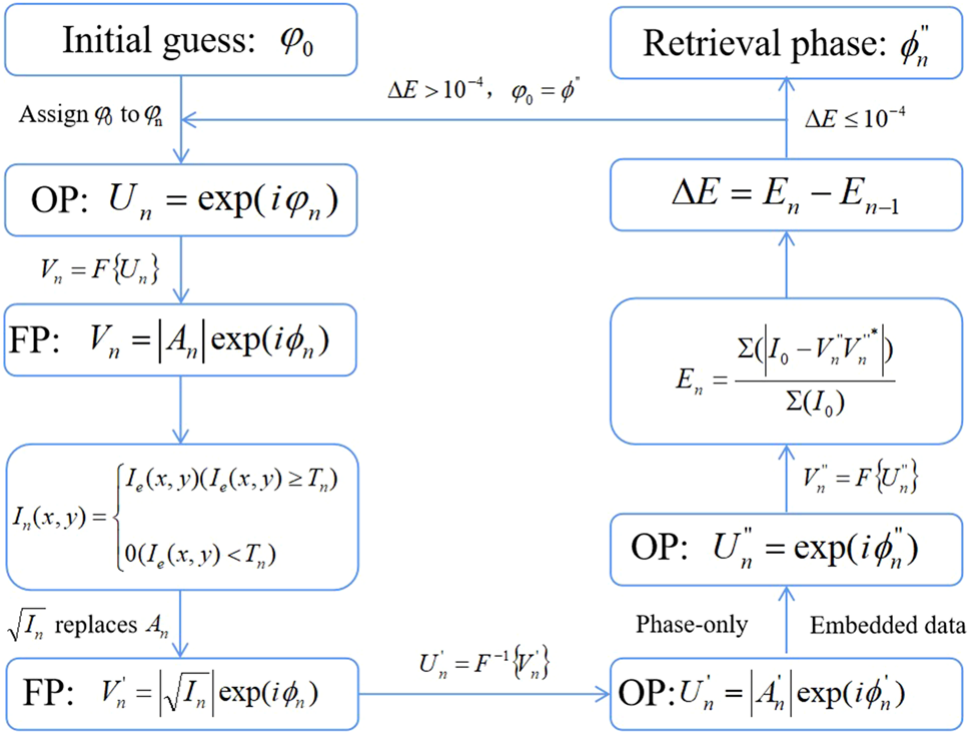
Flow diagram of IFTA for dynamic sampling.

Next, the new extended Fourier intensity is dynamically sampled. The dynamic sampling refers to the adaptive sampling strategy employed during different iterations of the phase retrieval process. It means that the sampling of the original spectrum varies based on the thresholds *T*_*n*_ at each iteration. The thresholds *T*_*n*_ determines the sampling rate for each iteration. By dynamically adjusting the sampling strategy based on the thresholds *T*_*n*_, the method can optimize the convergence of the phase retrieval algorithm. The flow diagram of IFTA for dynamic sampling is explained in Fig. 3. Firstly, an initial guess of the reconstructed phase φ_0_ is set as the beginning of the iteration, and then assign φ_0_ to φ_n_, where *n* = 1, 2, 3… denotes iterative number. So the initial guess complex amplitude distribution *U*_*n*_ in the object plane can be got. After Fourier transform, we can obtain a complex amplitude distribution *V*_*n*_ in the Fourier plane. Then, dynamic sampling is carried out for the intensity distribution of the expanded Fourier frequency *I*_*e*_. A series of thresholds *T*_*n*_ according to the discarded gray values in different iterations are set to provide a better convergence path for phase retrieval. We investigated the discarded gray values in more detail in another paper^27^. Next, the amplitude *A*_*n*_ is replaced by the square root of the sampled intensity distribution $\sqrt {I_{e} }$. In traditional IFTA, the amplitude *A*_*n*_ is always directly replaced by the square root of the intensity distribution captured by the detector. After inverse Fourier transform, the distribution in the object plane is got. The phase-only code and the embedded data as constraints can renew the distribution. Finally, we calculate the intensity error ratio *E*_*n*_ and the difference Δ*E* between two adjacent intensity error rates as the convergence condition. When the difference is less than 10^−4^, we think that retrieval phase is accurate. Otherwise, the iteration could be run until a convergence condition is met. The IFTA for dynamic sampling in this paper is to dynamically sample the expanded Fourier intensity image according to the threshold *T*_*n*_. By simulating the parameters of the experimental system, the training curve for the threshold *T*_*n*_ can be calculated and the extended Fourier intensity image of the experiment can be dynamically sampled based on the training curve.

In conclusion, we artificially complete the high-frequency information with the appropriate Nyquist size using the frequency expansion method, while only recording and capturing the Nyquist frequency. Subsequently, combining with the dynamic sampling method, the new extended Fourier intensity is sampled differently at various iteration times to achieve phase accelerated retrieval. The proposed method in this paper, which combines the frequency expanded method and the dynamic sampling method, offers faster phase retrieval for holographic data storage with improved storage density.

## Numerical simulation results

In simulation and experiment, each phase data is represented by 4 × 4 pixels on the SLM, and a single SLM pixel has dimensions 20 μm × 20 μm. To improve the convergence speed, embedded data with a 50% random distribution is used in IFTA, as traditional IFTA required hundreds of iterations. We use the frequency expanded method first to build a new spectrum owning more high-frequency components, and then use the dynamic sampling method to provide a faster convergence path for the phase retrieval. The phase data pages are generated with random 4-level phase patterns (0, π/2, π, 3π/2) for different image sizes (32 × 32, 64 × 64, 128 × 128, and 256 × 256 pixels).

We can get the 2*w*–5*w* expanded spectrum from Nyquist frequency by the frequency expanded method. Then, the SNR and BER of the phase retrieval results were calculated for comparison to find the optimal choice. In our simulations, the difference Δ*E* between two adjacent intensity error rates is less than 10^−4^, or circulation in the IFTA would stop after 100 iterations without convergence to 10^−4^. The low-frequency components with high power in the central region of the Fourier intensity image, which approximates the image information. By copying and pasting the normalized Nyquist frequency with its periodicity and assigning the corresponding intensity distribution, the high-frequency components can be approximately retrieved. Both noise-free and noisy simulations are performed. Firstly, the frequency expanded results without noise are represented in Table 1. The expanded Fourier intensity is obtained from the ideal Nyquist frequency, but it leads to the generation of error noise. It is important to note that the Fourier intensity distribution corresponding to the rectangular window in the simulation calculation is similar to the envelope of the actual Fourier intensity distribution, but not it is not an exact match. Therefore, error noise is introduced in the frequency expanded process. Next, a white Gaussian noise is added to reproduce the SNR of 8, which is similar to the experimental noise. The results are shown in Table 2. The Fourier intensity with Nyquist size, used for periodic expansion, primarily consists of low-frequency components with high power, offering better noise resistance compared to high-frequency components with lower power. Consequently, the error noise generated by the frequency expansion method is smaller than the actual noise, as demonstrated in Table 2, where the SNR for the expanded spectra of different sizes exceeds 8. Analysing the outcomes presented in both Tables 1 and 2, we can concluded that when the Fourier intensity with Nyquist size is expanded to 4 times Nyquist size, the SNR improves, and phase retrieval becomes more accurate with relatively few iterations. Generally, a larger expanded spectrum encompasses more high-frequency components, which are beneficial for precise phase retrieval. Therefore, compared to 3 times and 4 times expansion, the 2 times expansion results in higher BER values and lower imaging quality. However, the 5 times expansion yields worse results than 4 times expansion due to the poor noise resistance of high-frequency components and the introduction of error noise by the frequency expanded method. For instance, when the phase data page is a 256 × 256 data matrix, the BER for the retrieval phase, corresponding to the expanded Fourier intensity from Nyquist frequency to 5 times Nyquist frequency, can reach as high as nearly 50%. This phenomenon might be attributed to the intricate and noise-sensitive nature of the high-frequency spectrum distribution, leading to an excessive introduction of error noise through the frequency expansion process, which subsequently impacts the phase retrieval results negatively. Therefore, based on the comprehensive analysis of Tables 1 and 2, we conclude that expanding the original Nyquist frequency to 4 times Nyquist frequency is the most appropriate choice.

**Table 1:** In noise-free simulations, the results among the 2–5*w* expanded spectrum.

Phase data	The size of expanded spectrum	SNR	Iteration	BER (%)
32 × 32	2*w*	18.78	10	0
	3*w*	22.00	9	0
	4*w*	28.73	8	0
	5*w*	24.88	8	0
64 × 64	2*w*	18.60	14	0
	3*w*	21.65	11	0
	4*w*	27.04	9	0
	5*w*	23.33	12	0
128 × 128	2*w*	17.59	18	0
	3*w*	19.67	12	0
	4*w*	21.91	12	0
	5*w*	19.98	26	0
256 × 256	2*w*	14.21	100	0.40
	3*w*	16.09	45	0
	4*w*	16.81	23	0
	5*w*	14.21	100	46.81

**Table 2:** In the simulation with SNR of 8, the results among the 2–5*w* expanded spectrum.

Phase data	The size of expanded spectrum	SNR	Iteration	BER (%)
32 × 32	2*w*	11.47	13	0
	3*w*	11.87	9	0
	4*w*	12.17	8	0
	5*w*	12.02	8	0
64 × 64	2*w*	11.45	18	0
	3*w*	11.85	14	0
	4*w*	12.16	12	0
	5*w*	11.95	15	0
128 × 128	2*w*	11.25	100	0.50
	3*w*	11.61	23	0
	4*w*	11.85	20	0
	5*w*	11.64	100	4.48
256 × 256	2*w*	10.54	100	12.17
	3*w*	10.84	100	6.30
	4*w*	10.99	100	4.74
	5*w*	10.21	100	48.91

Considering that the expanded Fourier intensity with 4 times Nyquist size achieved the best result, it will serve as the basis for comparisons in the subsequent simulations and experiments. We carried out phase retrieval in different retrieval sources: (1) The Fourier intensity with 4 times Nyquist size is used directly; (2) The expanded Fourier intensity from Nyquist frequency to 4 times Nyquist frequency is used; (3) The dynamic sampling of the expanded 4 times Nyquist frequency is used.

**Figure 4:**
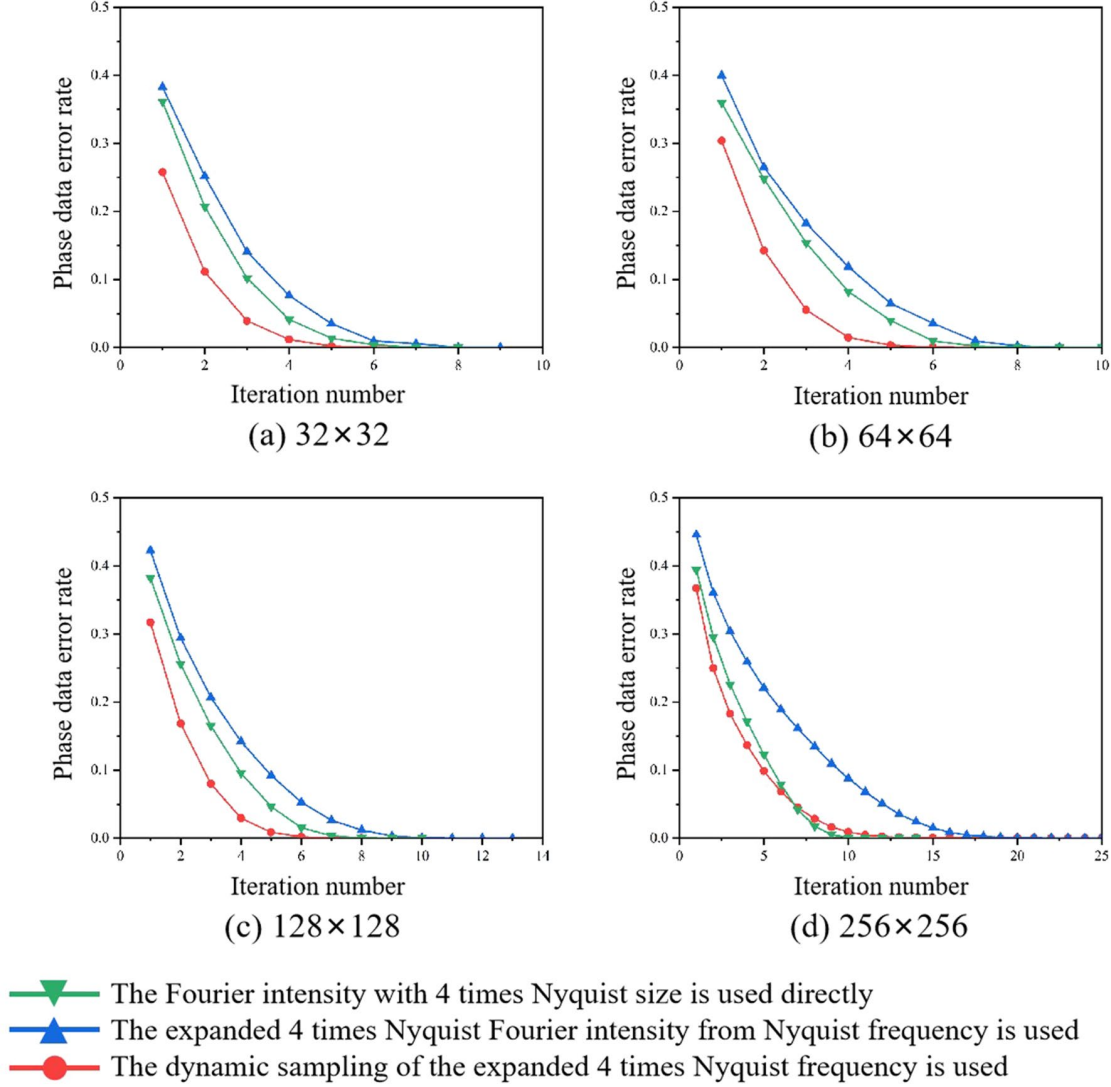
In noise-free simulations, the comparison of the phase retrieval results in the different retrieval sources. (**a**) 32 × 32, (**b**) 64 × 64, (**c**) 128 × 128, (**d**) 256 × 256.

Figure 4 shows the comparison of the phase retrieval results in the above three retrieval sources under ideal conditions. An ideal Fourier intensity does not contain any noise, and its phase retrieval result should be fast with a low BER. It is evident that the iterative number of the expanded Fourier intensity has increased compared to the ideal Fourier intensity with 4 times Nyquist size, due to the new error noise introduced by the frequency expanded method. Meanwhile the expanded Fourier intensity combined with the dynamic sampling method can reduce the iterative number, and the phase can be retrieved even faster in most cases. However, it is important to note that the frequency expanded method will introduce noise, especially the more complex Fourier spectrum are more sensitive to noise.

**Figure 5:**
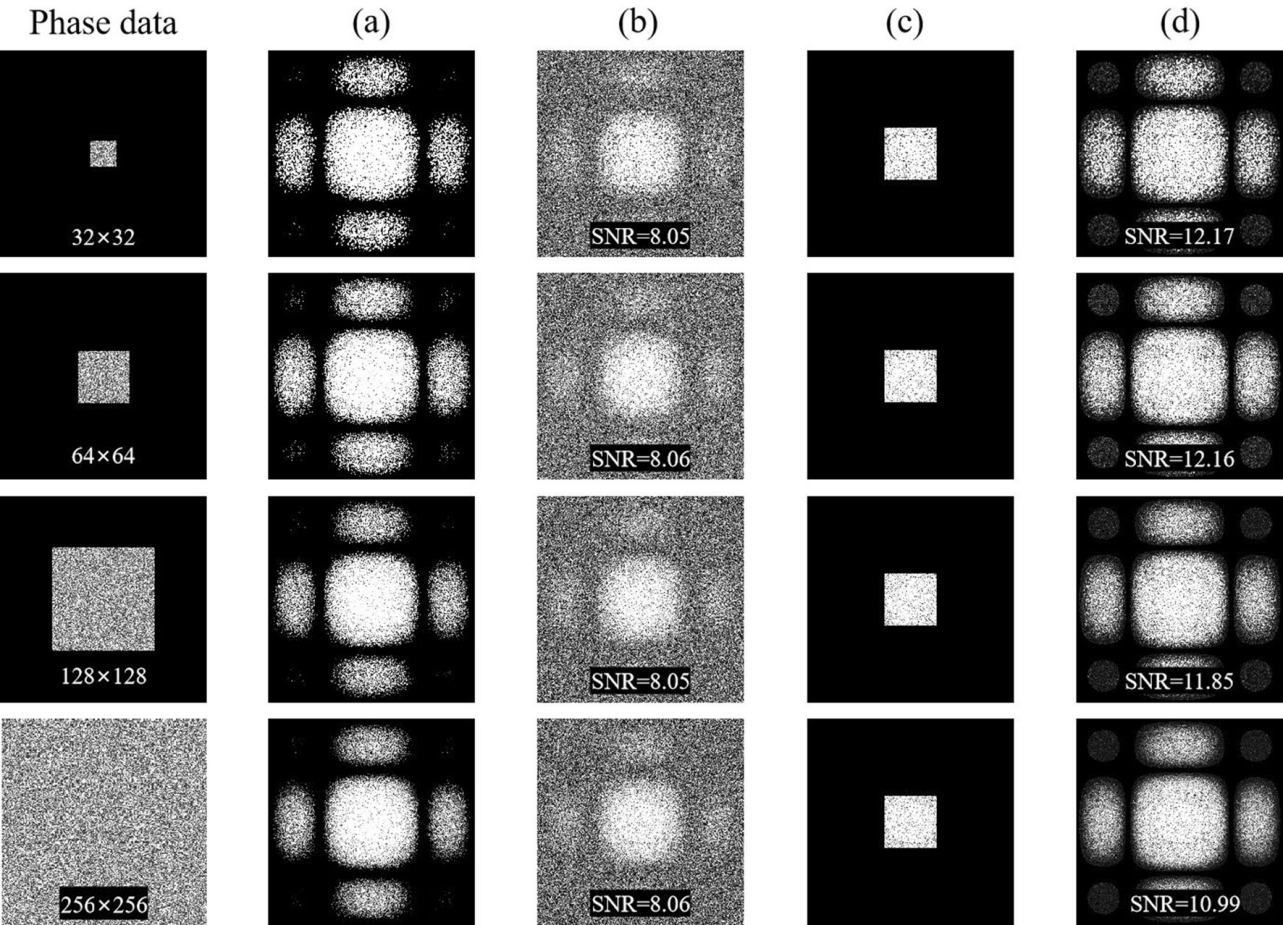
In the simulation, Signal-to-noise ratio of different Fourier intensity with 4 times Nyquist size. (**a**) the ideal Fourier intensity, (**b**) the Fourier intensity with noise, (**c**) the Fourier intensity with Nyquist size, (**d**) the expanded Fourier intensity from only Nyquist frequency of image (**c**).

**Figure 6:**
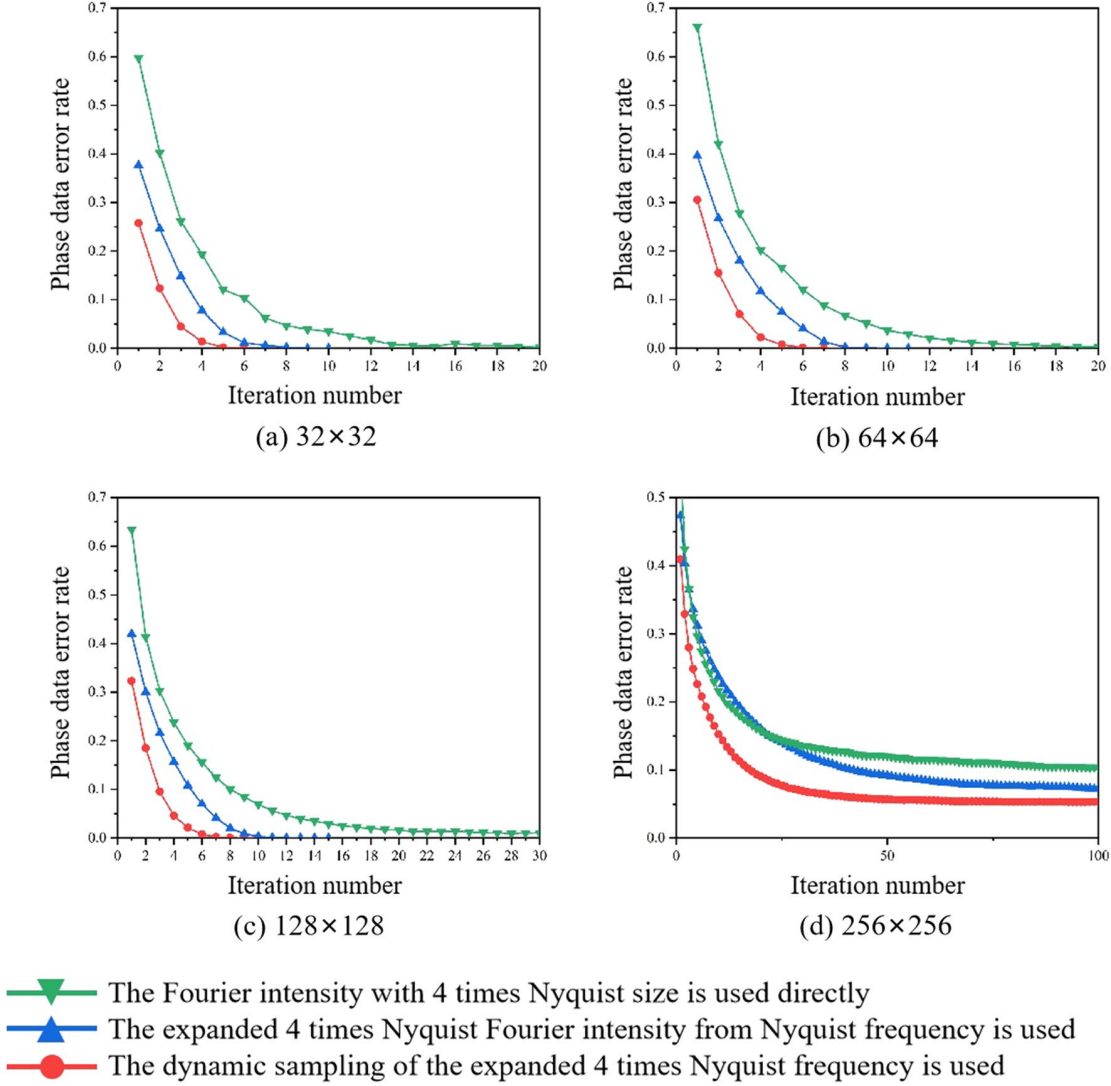
In the simulation with noise, the comparison of the phase retrieval results in the different retrieval sources. (**a**) 32 × 32, (**b**) 64 × 64, (**c**) 128 × 128, (**d**) 256 × 256.

In the simulations, we used a simplified noise distribution with an SNR level of 8, as illustrated in Fig. 5. To enhance clarity, we increased the image display intensity for better visualization. Figure 5a is the Fourier intensity with 4 times Nyquist size in the ideal case. Figure 5b introduces white Gaussian noise to the Fourier intensity in Fig. 5a, resulting in a Fourier intensity with noise. Figure 5d is the expanded Fourier intensity from only Nyquist frequency of Fig. 5c. The improvement in the distribution of the high-frequency component can be clearly seen by comparing Fig. 5b,d. And the SNR of Fig. 5d is higher than Fig. 5b. This can be attributed to the fact that the Fourier intensity spectrum at the Nyquist size contains essential information required for phase retrieval and exhibits relatively robust noise tolerance. The comparison of the phase retrieval results in the above three retrieval sources is shown in Fig. 6. The Fourier intensity with Nyquist size is composed of low-frequency components, which exhibit high power and strong resistance to noise. In the simulation with noise, the expanded 4 times Nyquist Fourier intensity from Nyquist frequency has a higher SNR and better phase retrieval results than the 4 times Nyquist Fourier intensity with a SNR of 8. Combining with the dynamic sampling method provides a faster convergence path for phase retrieval. When the matrix of the phase data page is larger, the corresponding Fourier spectrum is more complicated. This complexity poses a challenge for IFTA's phase retrieval capabilities, leading to higher BER, as shown in Fig. 6d. Results shown in Fig. 6 demonstrated that the phase retrieval of expanded spectrum by using dynamic sampling method is accurate, rapid and robust.

## Experimental results

**Figure 7:**
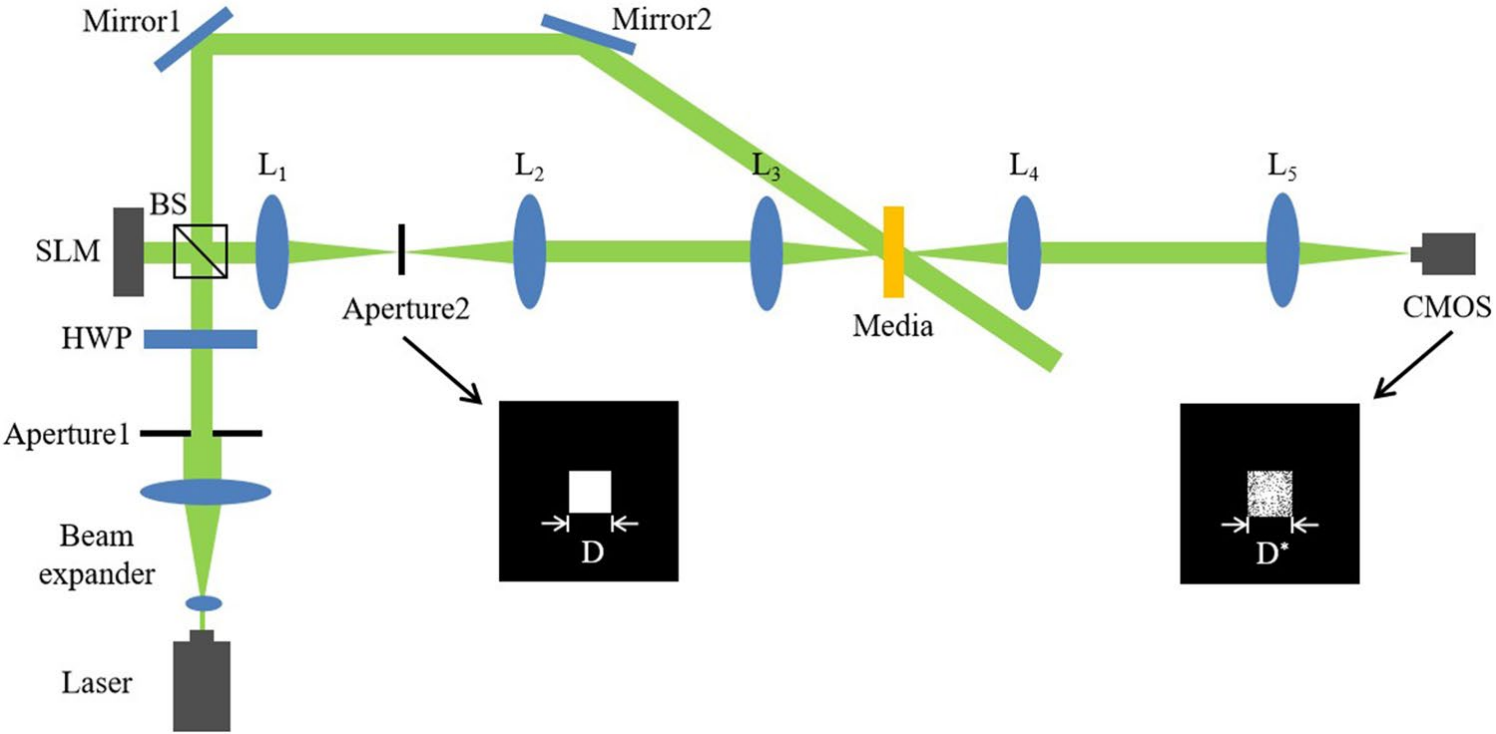
The optical setup of holographic data storage system. *HWP* half wave plate, *BS* beam splitter, *L1–L5* lens (L1–L5 = 150 mm), *SLM* spatial light modulator.

**Figure 8:**
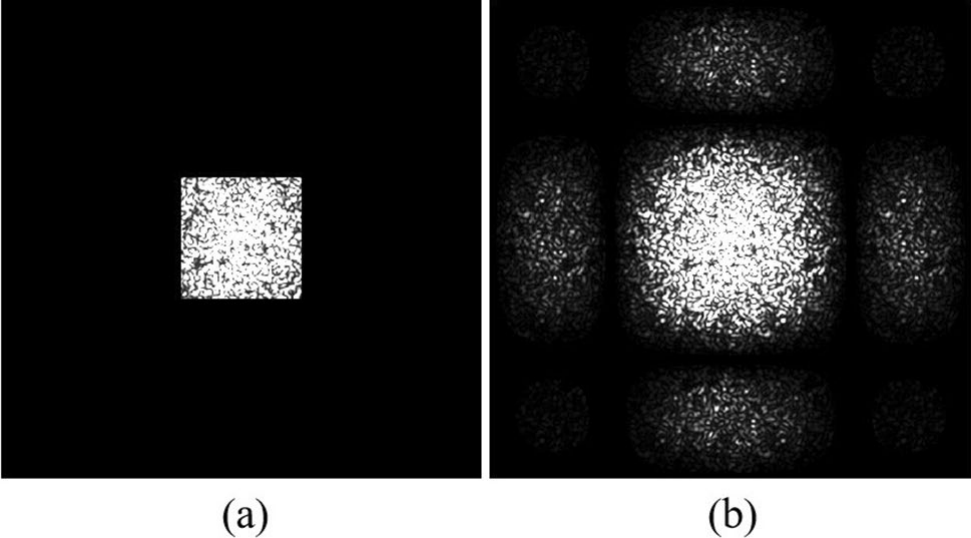
(**a**) The experimental Fourier intensity with Nyquist size captured by CMOS, (**b**) the expanded Fourier intensity from only Nyquist frequency captured by CMOS.

**Figure 9:**
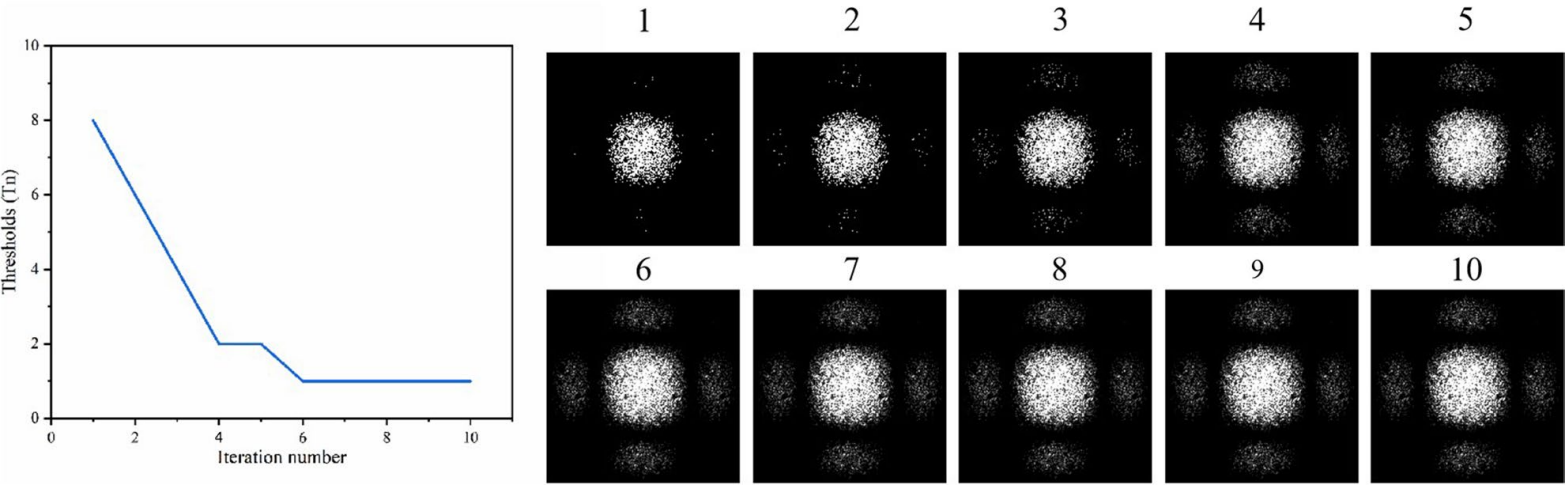
The training curve and first to tenth sampling intensity distributions for iteration calculation.

**Figure 10:**
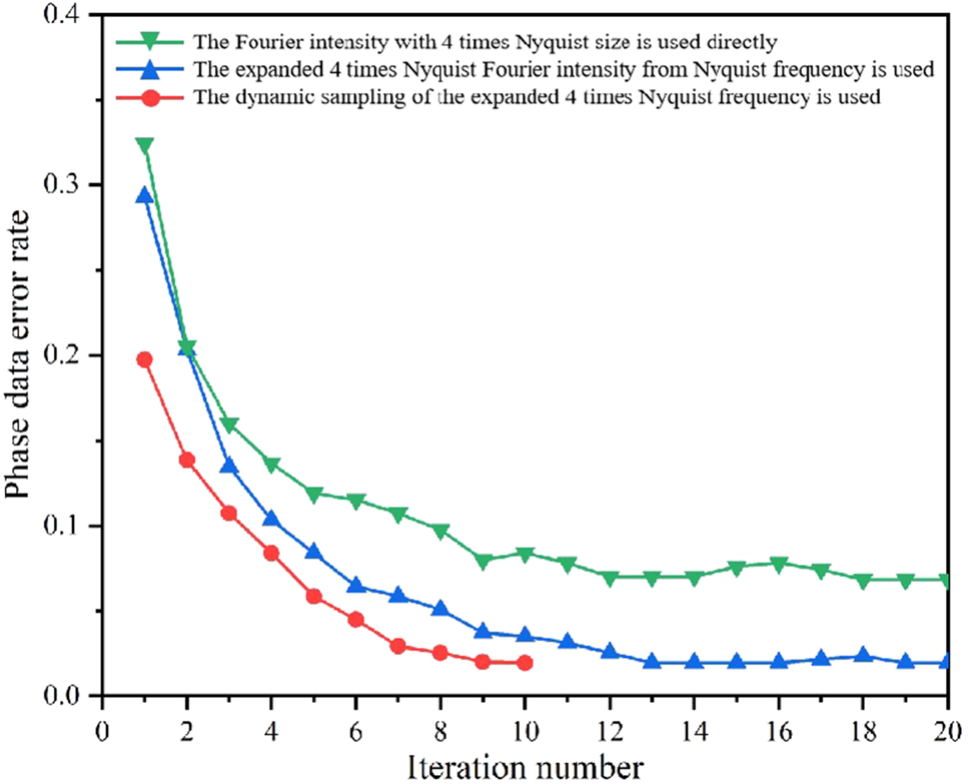
In the experiment, the comparison of the experimental phase retrieval results in the different retrieval sources.

**Figure 11:**
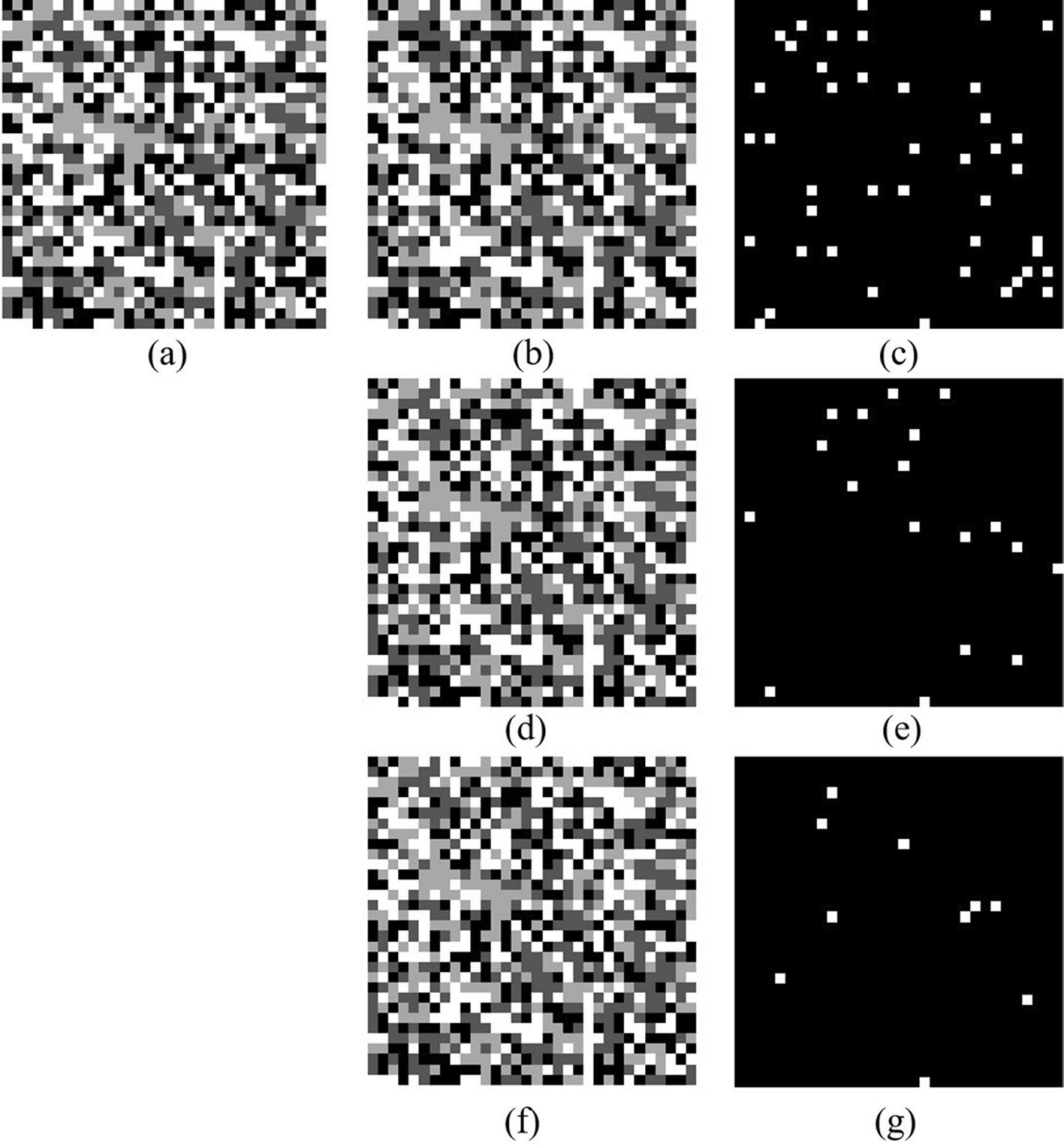
All retrieval results are got after 10 iterations. (**a**) The ground truth of phase data page, (**b**) the retrieval phase by the 4*w* spectrum direct as the retrieval source, (**c**) phase error distribution of (**b**), (**d**) the retrieval phase by the expanded 4*w* spectrum as the retrieval source, (**e**) phase error distribution of (**d**), (**f**) the retrieval phase by the dynamically sampled expanded spectrum as the retrieval source, (**g**) phase error distribution of (**f**).

The effectiveness of the proposed method is verified by experiments. The experimental setup is shown in Fig. 7. The light source is a 532 nm laser with an output power of 300 mW. We employ a phase-only SLM (X10468-04, Hamamatsu) with a resolution of 792 × 600 pixel and a pixel pitch of 20 μm. The CMOS (DCC1545M, Thorlabs) offer a fullframe resolution of 1280 × 1024 pixels with a pixel pitch of 5.2 μm. The phase data page is a 32 × 32 data matrix. Each phase data is represented by 4 × 4 pixels on the SLM, and a single SLM pixel has dimensions 20 μm × 20 μm, so the space width of one phase data in the SLM is 80 μm. The focal length of lens is 150 mm. The aperture size (D) is approximately 997.5 × 997.5 μm^2^ computed based on Eq. (1). The PQ/PMMA media is used with a thickness of 1 mm. Figure 8a is experimental Fourier intensity with Nyquist size captured by CMOS. The expanded 4 times Nyquist Fourier intensity from only Nyquist frequency captured by CMOS is shown in Fig. 8b. In the experiment, only Nyquist frequency recording is required, which will reduce media consumption and increase recording density. And the missing high-frequency information is artificially supplemented by the frequency expanded method. The data training is repeated multiple times to obtain a training curve in the simulation, and the input parameters are exactly the same as the system parameters. The specific training process is described in Ref.^26^, and thus is not covered in detail here. Then, the training curve is used to dynamically sample the expanded Fourier intensity, as shown in Fig. 9. Figure 10 shows the comparison of the experimental phase retrieval results in the different retrieval sources. The BER of the Fourier intensity with 4 times Nyquist size captured by CMOS is 6.84% after 20 iterations. The BER of the expanded Fourier intensity from only Nyquist frequency is 1.95% after 20 iterations. The dynamic sampling method can provide a better convergent path for the phase retrieval, the phase retrieval of expanded Fourier intensity by using dynamic sampling method only requires 10 iterations to achieve 1.95%. In summary, the proposed method can speed up the retrieval and reduce the BER. The retrieval phase and BER distribution results after 10 iterations in the different retrieval sources are shown in Fig. 11. Figure 11a is the ground truth of phase data page. Figure 11b,d,f are the retrieval phase by the 4*w* spectrum direct and the expanded 4*w* spectrum and the dynamically sampled expanded spectrum as the retrieval sources, respectively. Figure 11c,e,g are BER distribution of Fig. 11b,d,f, respectively. For the same number of iterations, the dynamically sampled expanded spectrum as the retrieval source achieves the best phase retrieval result. The simulation and experimental results demonstrate the possibility of integrating various methods on the Fourier domain and provide a potential way to improve the performance of holographic data storage systems.

## Conclusions

In this paper, we proposed a phase retrieval of expanded spectrum by using dynamic sampling method. The frequency expanded method only requires recording the frequency spectrum with Nyquist size and achieves periodic extension of the spectrum, thereby artificially supplementing high-frequency information. We analyze and discuss the SNR and phase retrieval results of the expanded Fourier intensities with different Nyquist sizes, selecting an appropriate expansion multiple. Then, dynamic sampling is applied to the expanded spectrum to provide a better convergence path for phase retrieval. Both simulation and experiment results validate the effectiveness of the proposed method, which improves the storage density and provides an expanded Fourier intensity image with a higher SNR than the captured Fourier intensity image with the same size. Additionally, our method significantly reduces the BER of phase retrieval and the iterative number by 2 times. This method harmoniously merges two independent techniques, both operating within the Fourier spectrum, and showcases the potential for integrating diverse methods in holographic data storage.

## Data Availability

All data generated during this study are included in supplementary information file.
